# Exploring core mental health symptoms among persons living with HIV: A network analysis

**DOI:** 10.3389/fpsyt.2023.1081867

**Published:** 2023-01-20

**Authors:** Shuyu Han, Yizhu Zhang, Xianxia Yang, Ke Li, Lili Zhang, Ying Shao, Jianhong Ma, Yan Hu, Zheng Zhu, Yukun Zhang, Zhiwen Wang

**Affiliations:** ^1^School of Nursing, Peking University, Beijing, China; ^2^School of Public Health, Wuhan University, Wuhan, Hubei, China; ^3^Department of Emergency, Peking University First Hospital, Beijing, China; ^4^Department of Nursing, Beijing Youan Hospital Affiliated with Capital Medical University, Beijing, China; ^5^Department of Infection, Beijing Youan Hospital Affiliated With Capital Medical University, Beijing, China; ^6^School of Nursing, Fudan University, Shanghai, China

**Keywords:** HIV, AIDS, mental health symptom, symptom management, network analysis

## Abstract

**Context:**

Persons living with HIV (PLWH) commonly experience mental health symptoms. However, little is known about the core mental health symptoms and their relationships.

**Objective:**

This study aimed to evaluate the prevalence of various mental health symptoms and to explore their relationships in symptom networks among PLWH.

**Methods:**

From April to July 2022, we recruited 518 participants through convenience sampling in Beijing, China, for this cross-sectional study. Forty mental health symptoms, including six dimensions (somatization symptoms, negative affect, cognitive function, interpersonal communication, cognitive processes, and social adaptation), were assessed through paper-based or online questionnaires. Network analysis was performed in Python 3.6.0 to explore the core mental health symptoms and describe the relationships among symptoms and clusters.

**Results:**

Of the 40 mental health symptoms, the most common symptoms were fatigue (71.2%), trouble remembering things (65.6%), and uncertainty about the future (64.0%). In the single symptom network, sadness was the most central symptom across the three centrality indices (r_S_ = 0.59, r_C_ = 0.61, r_B_ = 0.06), followed by feeling discouraged about the future (r_S_ = 0.51, r_C_ = 0.57, r_B_ = 0.04) and feelings of worthlessness (r_S_ = 0.54, r_C_ = 0.53, r_B_ = 0.05). In the symptom cluster network, negative affect was the most central symptom cluster across the three centrality indices (r_S_ = 1, r_C_ = 1, r_B_ = 0.43).

**Conclusion:**

Our study provides a new perspective on the role of each mental health symptom among PLWH. To alleviate the mental health symptoms of PLWH to the greatest extent possible and comprehensively improve their mental health, we suggest that psychological professionals pay more attention to pessimistic mood and cognitive processes in PLWH. Interventions that apply positive psychology skills and cognitive behavioral therapy may be necessary components for the mental health care of PLWH.

## 1. Introduction

Persons living with HIV (PLWH) have achieved a satisfactory life expectancy due to the development and popularization of antiretroviral therapy (ART) (1, 2). The Joint United Nations Programme on HIV/AIDS (UNAIDS) advocated that the final goal was not virologicalsuppression but rather improving the health-related quality of life of PLWH (3). However, PLWH commonly suffer from mental health symptoms, which have become one of the major barriers to achieving the “90-90-90-90” goal (4). The global depression prevalence among PLWH is greater than 30% (5). Mental health issues are even more severe in Chinese PLWH (6), with depression and anxiety noted in 61% and 43%, respectively (7). PLWH who are virologically suppressed have more severe mental health problems than the general population (8, 9). This dire situation may be associated with various factors, including but not limited to multiple symptoms, ART side effects, economic and medical burdens, social stigma and discrimination, and/or the loss of social support (10, 11). Given that mental health symptoms may lead to many negative consequences, such as engaging in risky behaviors, decreasing medication and medical follow-up adherence, and increasing mortality rates (12–14), it is necessary and urgent to pay more and continuous attention to the mental health issues of PLWH.

Although there have been many studies focusing on mental health issues in PLWH, including prevalence evaluations (15–17), associated factor exploration (18, 19), intervention development and applications (20, 21), most previous studies have focused on a single mental health symptom. Other important dimensions of mental health symptoms, such as somatization symptoms, cognitive process symptoms, and social adaptation symptoms, are seldom comprehensively assessed. Moreover, previous studies more frequently studied mental health symptoms from a common cause perspective (22). More studies from the causal systems perspective, i.e., describing the possibility that symptom co-occurrence is due to direct symptom-to-symptom relationships rather than a common cause, are needed to help understand the mechanisms and relationships among mental health symptoms (23).

Network analysis can be applied to examine complex associations between symptoms and may help explain unanswered questions of comorbidities at the symptom level from the causal systems perspective (24, 25). To our knowledge, only one study has described the mental health symptom network for PLWH (26). However, this study only included 16 symptoms, which may not be sufficient to comprehensively understand the complex relationships of mental health symptoms for PLWH. Therefore, we aimed to extend the mental health symptom network analysis study using a 40-item mental health symptoms questionnaire that contains six dimensions (somatization symptoms, negative affect, cognitive function, interpersonal communication, cognitive processes, and social adaptation), thus identifying the core symptoms and describing the complex symptom relationships in the network. This attempt may provide a theoretical reference to understand the relationships of mental health symptoms among PLWH and guide the development of more precise and effective interventions to help improve the mental health of PLWH.

## 2. Methods

### 2.1. Setting and study participants

This study was conducted according to the World Medical Association Declaration of Helsinki and approved by the Peking University Biomedical Ethics Committee (IRB00001052-21055). From April 2022 to July 2022, we recruited participants in Beijing Youan Hospital Affiliated with Capital Medical University using convenience sampling. Two clinical nurses were trained to collect data *via* paper-based questionnaires and verify participants' clinical information in the hospital's medical record databases. They explained the study objectives and procedures to the participants face-to-face and obtained their informed consent from them. Participants usually spent 7–10 min completing the questionnaires and received 10 RMB as compensation. The inclusion criteria included (1) being diagnosed with HIV-1 infection; (2) being aged 18 years or older; (3) having at least one visit to Youan Hospital and having clinical data available in the databases; and (4) signing the informed consent form. The exclusion criteria were as follows: (1) the inability to complete the questionnaire by themselves for any reason, including but not limited to illiteracy, being blind or deaf, and severe comorbidities, such as various opportunistic infections and HIV-associated neurocognitive disorder (HAND); and (2) taking part in other HIV-related research projects simultaneously during our research. Ultimately, 518 PLWH participated in our study, 15 of whom (2.9%) were excluded because of missing data.

### 2.2. Measures

#### 2.2.1. Demographic and clinical characteristics

The demographic variables in the questionnaire included sex, age, ethnicity, religion, educational attainment, marital status, employment status, and monthly income. Clinical variables included monthly income, transmission mode, months since HIV diagnosis, CD4+ T-cell count, viral load, and comorbidities.

#### 2.2.2. Mental health symptoms

A total of 40 items were used to assess mental health in the questionnaire. Each item score ranged from 0 to 3. Higher scores indicated more severe mental health symptoms. According to the concept analysis of mental health (27, 28), mental health refers to the normal state of mental state, mental process, and mental activity. We assessed six dimensions for mental health symptoms, including somatization symptoms and negative affect to reflect metal state; cognitive processes and cognitive function to reflect mental process; interpersonal communication and social adaptation to reflect mental activity. The somatization subscale and interpersonal sensitivity subscales of the Brief Symptom Inventory (BSI-53) were applied to evaluate somatization symptoms and interpersonal communication, including seven items and four items, respectively (29). The 10-item negative affect subscale of the Positive and Negative Affect Schedule (PANAS) was applied to assess negative affect symptoms (30). Cognitive function symptoms were measured by five items from the subscale of the AIDS Health Assessment Questionnaire (AIDS-HAQ) (31). Three items were used to evaluate the social adaptation dimension, including escaping from reality, being unable to integrate into society, and being unable to handle daily work and study, which were also applied in our previous study (32). Cognitive processes were measured by seven items, including uncertainty about the future, feeling discouraged about the future, the belief that one should be punished for their sins, a lack of confidence around others, feeling that one is being watched or talked about by others, feelings of worthlessness, and suicidal ideation. Four additional mental health symptoms were included, including sleep satisfaction, loneliness, sadness, and anger.

### 2.3. Data analysis

We used SPSS 24.0 and Python 3.6.0 for data analysis. Descriptive analysis used the mean and standard deviation (SD) for continuous variables and frequencies and percentages for categorical variables. The Cronbach'α coefficient was used to evaluate the internal consistency of the mental health symptom subscales in our recruited participants. Network analysis was performed to describe the relationships among mental health symptoms and symptom clusters. In the network analysis, the Spearman correlation was applied to estimate the correlation relationships between the nodes in the networks; the Fruchterman-Reingold algorithm was applied to place nodes with the strongest correlations at the center of the network (33). Centrality indices, including strength, closeness, and betweenness, were used to identify the most central symptoms and symptom clusters in the networks (34). All the centrality indices were standardized (reporting r between 0 and 1) to make the nodes more comparable.

## 3. Results

Table 1 shows the demographic and clinical characteristics of the participants. The majority of the participants were male (99.0%), of Han ethnicity (92.0%), non-religious (89.3%), and single (72.2%). The average age of the participants was 35.73 years. Nearly 70% of the participants had obtained a bachelor's degree or above. Approximately half of the participants (56.7%) earned more than 5000 RMB per month. Only 45.9% of the participants had formal jobs. The main transmission mode was sexual behavior (68.0%). The average time since HIV diagnosis was 79.94 months. The mean CD4+ T-cell count was 564/μL. Most of the participants had undetectable viral loads (91.2%) and did not have comorbidities.

**Table 1:** Participant characteristics (*N* = 503).

**Characteristics**	***N* (%), M ±SD**
**Sex**	
Male	498 (99.0)
Female	5 (1.0)
**Age**	35.73 ± 8.60
**Ethnicity**	
Han	463 (92.0)
Minority	40 (8.0)
**Religion**	
Yes	54 (10.7)
No	449 (89.3)
**Educational attainment**	
Middle school or below	48 (9.5)
Senior high school	105 (20.9)
University	302 (60.0)
Master's degree or above	48 (9.5)
**Marital status**	
Single	363 (72.2)
Married	91 (18.1)
Divorced	46 (9.1)
Widowed	3 (0.6)
**Employment status**	
Employed	231 (45.9)
Self-employed	227 (45.1)
Unemployed	45 (9.0)
**Monthly income**	
2500 RMB or below	44 (8.7)
2500 RMB - 5000 RMB	174 (34.6)
5000 RMB or above	285 (56.7)
**Transmission mode**	
Blood	18 (3.6)
Sexual behavior	342 (68.0)
Unknown	143 (28.4)
**Months since HIV diagnosis**	79.94 ± 45.21
**CD4+** **T-cell count**	563.5 ± 267.40
**Viral load**	
Detectable	38 (8.8)
Undetectable	393 (91.2)
**Comorbidity**	
Yes	56 (11.1)
No	447 (88.9)

As presented in Table 2, all six subscales showed satisfactory Cronbach's α values, which were all >0.85. The top three prevalent mental health symptom clusters were somatization symptoms (78.5%), negative affect (75.9%), and cognitive function (72.2%). Of the 40 mental health symptoms, the most common symptoms were feeling weakness in parts of the body (71.2%), forgetting things that occurred recently (65.6%), uncertainty about the future (64.0%), sleep disturbance (59.2%), and nervousness (59.2%). The most severe symptom was uncertainty about the future, followed by feeling weakness in parts of the body, forgetting things that occurred recently, sleep disturbance, and loneliness.

**Table 2:** Prevalence and severity of mental health symptoms (*N* = 503).

**Mental health symptoms**	**Prevalence, *N* (%)**	**Severity (Mean ±SD)**	**Cronbach's Alpha**
**Somatization symptom**	394 (78.5)	3.49 ± 3.72	0.868
Faintness or dizziness	194 (38.6)	0.50 ± 0.73	
Pain in the heart or chest	136 (27.0)	0.34 ± 0.63	
Nausea or upset stomach	89 (17.7)	0.23 ± 0.56	
Trouble catching your breath	152 (30.2)	0.41 ± 0.71	
Hot or cold spells	163 (32.6)	0.45 ± 0.73	
Numbness or tingling in parts of your body	201 (40.0)	0.53 ± 0.75	
Feeling weak in parts of your body	358 (71.2)	1.02 ± 0.85	
**Cognitive function**	363 (72.2)	3.48 ± 3.55	0.921
Difficulty concentrating	258 (51.3)	0.75 ± 0.86	
Forgetting things that occurred recently	330 (65.6)	0.94 ± 0.86	
Difficulty reasoning	186 (37.0)	0.50 ± 0.76	
Becoming confused	183 (36.4)	0.52 ± 0.79	
Having slow reactions	282 (56.1)	0.77 ± 0.81	
**Negative affect**	382 (75.9)	7.25 ± 7.54	0.953
Distressed	206 (41.0)	0.63 ± 0.89	
Upset	284 (56.5)	0.84 ± 0.91	
Guilty	224 (44.5)	0.71 ± 0.94	
Scared	233 (46.3)	0.72 ± 0.92	
Hostile	148 (29.4)	0.42 ± 0.75	
Irritable	268 (53.3)	0.81 ± 0.91	
Ashamed	183 (36.4)	0.56 ± 0.88	
Nervous	296 (58.8)	0.88 ± 0.91	
Jittery	269 (53.5)	0.81 ± 0.92	
Afraid	233 (46.3)	0.72 ± 0.92	
**Interpersonal communication**	303 (60.2)	2.45 ± 2.90	0.899
Your feelings are easily hurt	234 (46.5)	0.66 ± 0.83	
Feeling that people are unfriendly or dislike you	178 (35.4)	0.47 ± 0.74	
Feeling inferior to others	227 (45.1)	0.68 ± 0.90	
Feeling very self-conscious around others	232 (46.1)	0.65 ± 0.84	
**Cognitive processes**	351 (69.8)	4.45 ± 5.04	0.915
Uncertainty about the future	322 (64.0)	1.07 ± 1.01	
Feeling discouraged about the future	247 (49.1)	0.79 ± 0.96	
The belief that I should be punished for my sins	148 (29.4)	0.44 ± 0.79	
Lack of confidence around others	216 (42.9)	0.65 ± 0.88	
Feeling that I am being watched or talked about by others	145 (28.8)	0.39 ± 0.70	
Feelings of worthlessness	203 (40.4)	0.61 ± 0.87	
Suicidal ideas	156 (31.0)	0.49 ± 0.85	
**Social adaptation**	231 (45.9)	1.43 ± 2.13	0.880
Escaping from reality	212 (42.1)	0.63 ± 0.87	
Unable to integrate into society	154 (30.6)	0.46 ± 0.80	
Unable to handle daily work and study	120 (23.9)	0.34 ± 0.69	
**Other symptoms**	—	—	—
Sleep disturbance	298 (59.2)	0.92 ± 0.93	
Loneliness	285 (56.7)	0.90 ± 0.98	
Sadness	240 (47.7)	0.78 ± 0.98	
Anger	189 (37.6)	0.56 ± 0.85	

**Figure 1:**
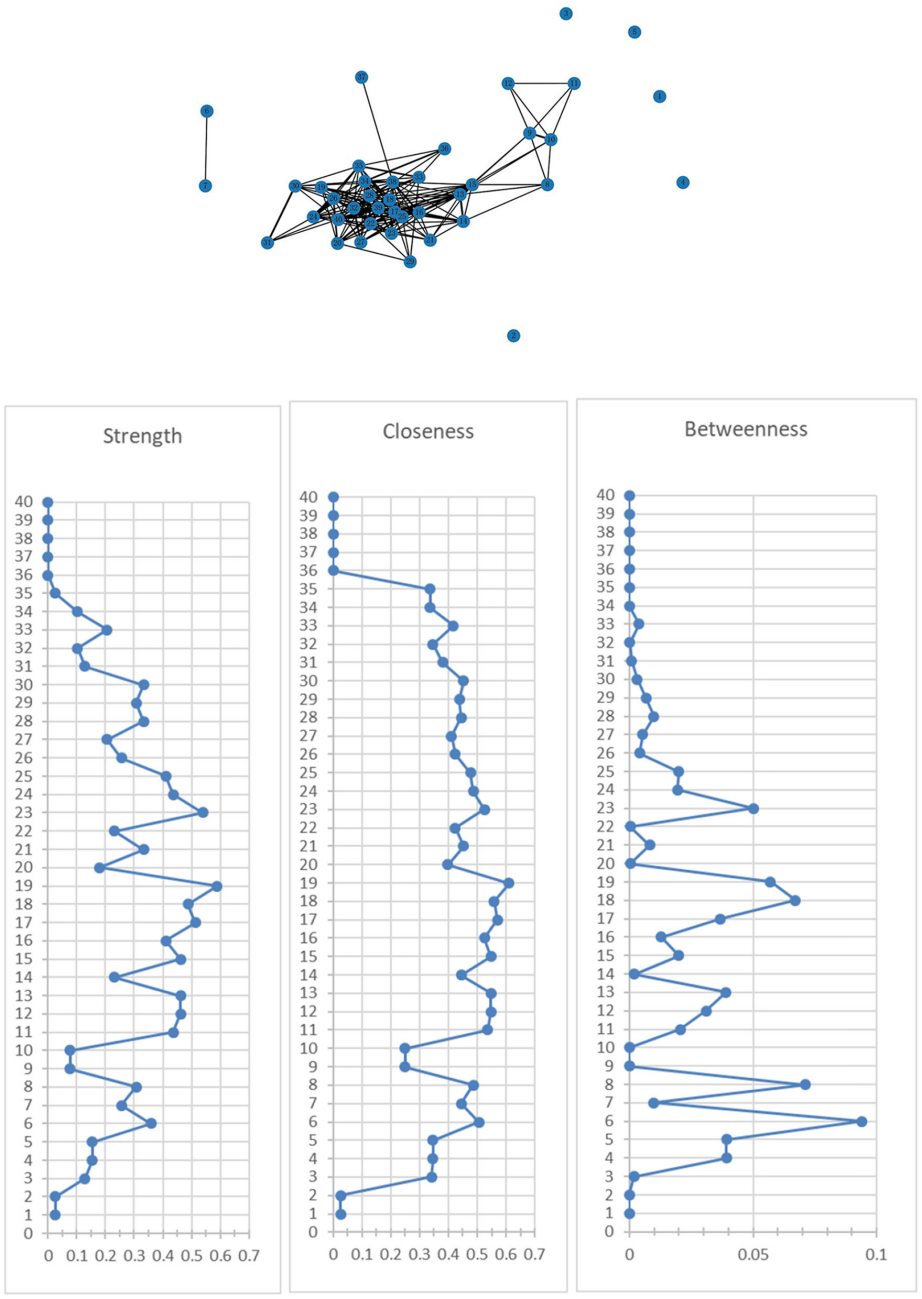
Network of mental health symptoms and centrality indices. 1. Feeling weak in parts of your body; 2. Faintness or dizziness; 3. Hot or cold spells; 4. Numbness or tingling in parts of your body; 5. Nausea or upset stomach; 6. Pain in the heart or chest; 7. Trouble catching your breath; 8. Difficulty concentrating; 9. Having slow reactions; 10. Forgetting things that occurred recently; 11. Difficulty reasoning; 12. Becoming confused; 13. Jittery; 14. Nervous; 15. Afraid; 16. Upset; 17. Distressed; 18. Guilty; 19. Hostile; 20. Ashamed; 21. Irritable; 22. Scared; 23. Your feelings are easily hurt; 24. Feeling that people are unfriendly or dislike you; 25. Feeling inferior to others; 26. Feeling very self-conscious around others; 27. Uncertainty about the future; 28. Feeling discouraged about the future; 29. The belief that I should be punished for my sins; 30. Lack of confidence around others; 31. Feeling that I am being watched or talked about by others; 32. Feelings of worthlessness; 33. Suicidal ideas; 34. Escaping from reality; 35. Unable to integrate into society; 36. Unable to handle daily work and study; 37. Sleep disturbance; 38. Loneliness; 39. Sadness; 40. Anger.

Figure 1 shows the association network and centrality indices among the 40 mental health symptoms. The top three strongest edges were between “nervousness” and “jittery” (*r* = 0.85), “loneliness” and “sadness” (*r* = 0.84), and “uncertainty about the future” and “feeling discouraged about the future” (*r* = 0.81). In the entire network, “sadness” (r_S_ = 0.59, r_C_ = 0.61, r_B_ = 0.06) was the most central symptom across the three centrality indices, followed by “feeling discouraged about the future” (r_S_ = 0.51, r_C_ = 0.57, r_B_ = 0.04) and “feeling of worthlessness” (r_S_ = 0.54, r_C_ = 0.53, r_B_ = 0.05).

**Figure 2:**
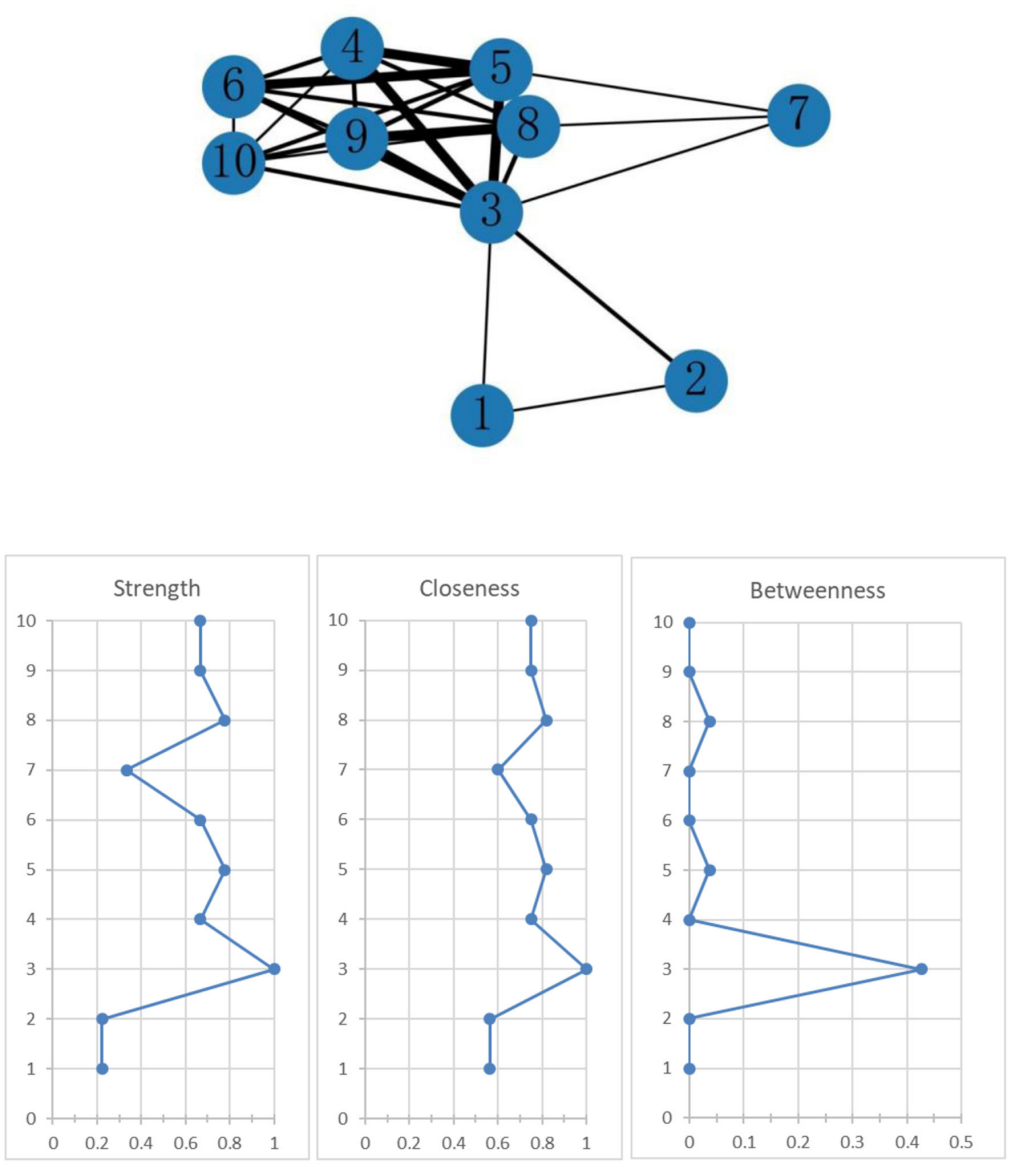
Network of mental health symptom clusters and centrality indices. 1. Somatization symptom cluster; 2. Negative affect symptom cluster; 3. Cognitive function symptom cluster; 4. Interpersonal communication symptom cluster; 5. Cognitive process symptom cluster; 6. Social adaptation symptom cluster; 7. Sleep disturbance; 8. Loneliness; 9. Sadness; 10. Anger.

Figure 2 shows the association network and centrality indices among the six mental health symptom clusters and four mental health symptoms. In the entire network, the top three strongest edges were between “loneliness” and “sadness” (*r* = 0.84), “negative affect” and “cognitive processes” (*r* = 83), and “interpersonal communication” and “cognitive processes” (*r* = 0.82). In the entire network, “negative affect” (r_S_ = 1, r_C_ = 1, r_B_ = 0.43) was the most central symptom cluster across the three centrality indices, followed by “cognitive processes” (r_S_ = 0.78, r_C_ = 0.82, r_B_ = 0.04).

## 4. Discussion

Our study contributes to the current literature on the comprehensive assessment of mental health symptoms, exploring core symptoms and symptom clusters through network analysis among PLWH. The top three most prevalent mental health symptoms were weakness in parts of the body, trouble remembering things, and uncertainty about the future. The top three most central symptoms in the network were sadness, feeling discouraged about the future, and feelings of worthlessness. Of the six mental health symptom dimensions, negative affect was the most central symptom cluster.

The results indicated that PLWH experienced prevalent and multiple mental health symptoms. Parcesepe et al. reported that the prevalence of psychiatric comorbidities among PLWH was as high as 42% (35). In our study, eleven of the forty symptoms had a prevalence of more than 50%. All six symptom clusters showed a prevalence of more than 45%, which hinted at the necessity and urgency of mental health service implementation and a comprehensive view of mental health symptoms rather than focusing on only a single or several symptoms. Both the symptom network and symptom cluster network presented a large network density, which indicated a high probability of mental health symptom comorbidity and emotional neuroticism. This phenomenon also suggested the importance of intervening in core mental health symptoms to improve the efficiency and effectiveness of interventions (36, 37).

Of the 40 investigated mental health symptoms, feeling weak in parts of your body, forgetting things that occurred recently, uncertainty about the future, and sleep disturbance showed the top four highest incidences and severity. Previous studies also showed these symptoms were prevalent among PLWH. For instance, the incidence of fatigue and memory loss was reported to be ~50% among PLWH (38–40). The incidence of sleep disturbance was as high as 57.6–75% among PLWH (41, 42). PLWH also reflected multiple reasons for uncertainty about the future, such as health challenges, financial uncertainty, symptom distress, caregiver uncertainty, and medical uncertainty (43, 44). Moreover, interventions for these symptoms usually did not consider their correlations with other symptoms and tended to use comprehensive strategies. Fatigue management strategies for PLWH include behavioral activation counseling, exercise, diet intervention, and relaxation activities (45, 46). Physical activity, medication therapy, and adjunctive therapy are common strategies for cognitive symptom management (47, 48). Sleep management strategies for PLWH include sleep hygiene training and medication therapy (49).

Previous symptom management usually pays more attention to the more prevalent symptoms or treats PLWH's multiple symptoms as an integrated problem (50–52). However, the most common symptoms or most severe symptoms were usually different from the most central symptoms in the network analysis. Zhu et al. described the contemporaneous symptom network for PLWH. Of the 27 symptoms, fatigue was the most common and severe symptom, while “feeling nervous” was the most central symptom in the network (53). In our study, the top three most central symptoms were not the most common or severe symptoms. These results remind researchers and clinical practitioners to reconsider the priorities of clinical practice according to the causal systems perspective. If mental health interventions are designed according to the prevalence and/or severity of mental health symptoms and ignore the interconnection between different symptoms, these interventions may not be the most cost-effective approaches to improve the mental health of PLWH. A previous study reported “sadness”, “panic”, and “self-abasement” were the top three most central psychological symptoms (26). While in this study, the top three most central symptoms among our participants were “sadness”, “feeling discouraged about the future”, and “feeling of worthlessness”. These results highlight that researchers and clinical practitioners should understand the importance of the influence of pessimistic mood and cognitive processes caused by HIV infection on the mental health of PLWH. Psychological skills that target these central mental health symptoms and can promote positive moods and cognitive processes, such as positive psychology skills and cognitive behavioral therapy, may be necessary components for the mental health care of PLWH (54, 55).

In the symptom cluster network, “negative affect” was the most central symptom cluster across the three centrality indices, which did not report in a similar previous study (26). These results indicated that clinical practitioners should pay attention to helping PLWH improve negative moods. Previous studies indicated that PLWH showed a huge unmet need for emotional support (56, 57). Timely and effective regulation of negative moods may be more efficient in helping regulate other mental health symptom dimensions, such as cognitive process symptoms and social adaptation symptoms. The catharsis of negative emotions requires clinical practitioners to help PLWH appeal to their emotions, apply listening and reflective techniques, and demonstrate care, empathy, and comfort in an atmosphere of acceptance (58–60). Moreover, peer volunteers who are also PLWH may be more effective in empathizing with and helping PLWH vent their negative moods because of their similar experiences, which have irreplaceable advantages in the context of HIV-related discrimination (61).

Several limitations of this study should be noted. First, the cross-sectional design determines that we can only describe the unidirectionally contemporaneous mental health symptom networks, which makes it difficult to explore causal relationships. Dynamic network exploration using longitudinal data is needed in the future to provide insights into potential predictive associations between mental health symptoms. Second, the convenience sampling and single-center study setting make the sociodemographic characteristics of our participants difficult to generalize to the population of PLWH in China. Third, the small sample size makes it difficult to conduct subgroup analysis. Future studies with larger sample sizes are needed to compare mental health symptom network centrality indices among different demographic groups.

## 5. Conclusion

This study generated new knowledge about mental health symptom networks in PLWH. We found that sadness, feeling discouraged about the future, and feelings of worthlessness were the top three most central symptoms in the network. Of the six mental health symptom dimensions, negative affect was the most central symptom cluster. Our study suggests that psychological professionals should emphasize the significant influence of pessimistic mood and cognitive processes on the mental health of PLWH. These core symptoms may lead be pivotal breakthroughs for designing precise interventions and improving the mental health of PLWH. Psychological skills that target these core mental health symptoms, such as positive psychology therapy and cognitive behavioral therapy, may be necessary components for the mental health care of PLWH.

## Data availability statement

The raw data supporting the conclusions of this article will be made available by the authors, without undue reservation.

## Ethics statement

The studies involving human participants were reviewed and approved by the Peking University Biomedical Ethics Committee. The patients/participants provided their written informed consent to participate in this study.

## Author contributions

SH and ZZ participated in the research design. SH, YiZ, and XY drafted the manuscript. LZ, YS, JM, YH, and YuZ contributed to the acquisition of data. YiZ, XY, and KL performed the data analysis. ZW reviewed and edited the manuscript. All authors contributed to the article and approved the submitted version.

## References

[1] Antiretroviral Therapy Cohort Collaboration. Survival of HIV-positive patients starting antiretroviral therapy between 1996 and 2013: a collaborative analysis of cohort studies. Lancet HIV. (2017) 4:e349–56. 10.1016/S2352-3018(17)30066-8 28501495 PMC5555438

[2] Yoshimura K. Current status of HIV/AIDS in the ART era. J Infect Chemother. (2017) 23:12–6. 10.1016/j.jiac.2016.10.002 27825722

[3] Lazarus JV Safreed-Harmon K Barton SE Costagliola D Dedes N Del Amo Valero J. Beyond viral suppression of HIV – the new quality of life frontier. BMC Med. (2016) 14:94. 10.1186/s12916-016-0640-4 27334606 PMC4916540

[4] Li Y Guo Y Hong YA Zeng C Zeng Y Zhang H. Mediating effects of stigma and depressive symptoms in a social media-based intervention to improve long-term quality of life among people living with HIV: secondary analysis of a randomized controlled trial. J Med Internet Res. (2021) 23:e27897. 10.2196/27897 34751654 PMC8663519

[5] Rezaei S Ahmadi S Rahmati J Hosseinifard H Dehnad A Aryankhesal A. Global prevalence of depression in HIV/AIDS: a systematic review and meta-analysis. BMJ Support Palliat Care. (2019) 9:404–12. 10.1136/bmjspcare-2019-001952 31537580

[6] Wang T Fu H Kaminga AC Li Z Guo G Chen L. Prevalence of depression or depressive symptoms among people living with HIV/AIDS in China: a systematic review and meta-analysis. BMC Psychiatry. (2018) 18:160. 10.1186/s12888-018-1741-8 29855289 PMC5984474

[7] Niu L Luo D Liu Y Silenzio VMB Xiao S. The mental health of people living with HIV in China, 1998-2014: a systematic review. PLoS ONE. (2016) 11:e0153489. 10.1371/journal.pone.0153489 27082749 PMC4833336

[8] Drewes J Gusy B Rüden U. More than 20 years of research into the quality of life of people with HIV and AIDS–a descriptive review of study characteristics and methodological approaches of published empirical studies. J Int Assoc Provid AIDS Care. (2013) 12:18–22. 10.1177/1545109712456429 23042792

[9] Huang Y Luo D Chen X Zhang D Huang Z Xiao S. Role of psychosocial status in predicting health-related quality of life at 1-year follow-up among newly diagnosed people living with HIV. PLoS ONE. (2019) 14:e0224322. 10.1371/journal.pone.0224322 31644606 PMC6808448

[10] Brandt C Zvolensky MJ Woods SP Gonzalez A Safren SA O'Cleirigh CM. Anxiety symptoms and disorders among adults living with HIV and AIDS: a critical review and integrative synthesis of the empirical literature. Clin Psychol Rev. (2017) 51:164–84. 10.1016/j.cpr.2016.11.005 27939443 PMC5195877

[11] Van Der Heijden I Abrahams N Sinclair D. Psychosocial group interventions to improve psychological well-being in adults living with HIV. Cochrane Database Syst Rev. (2017) 3:CD010806. 10.1002/14651858.CD010806.pub2 28291302 PMC5461871

[12] Betancur MN Lins L Oliveira IRde Brites C. Quality of life, anxiety and depression in patients with HIV/AIDS who present poor adherence to antiretroviral therapy: a cross-sectional study in Salvador, Brazil. Braz J Infect Dis. (2017) 21:507–14. 10.1016/j.bjid.2017.04.004 28535397 PMC9425484

[13] Catalan J Tuffrey V Ridge D Rosenfeld D. What influences quality of life in older people living with HIV? AIDS Res Ther. (2017) 14:22. 10.1186/s12981-017-0148-9 28400851 PMC5387225

[14] Croxford S Miller RF Post FA Harding R Lucas SB Figueroa J. Cause of death among HIV patients in London in 2016. HIV Med. (2019) 20:628–33. 10.1111/hiv.12761 31274241

[15] Rahmati J Ahmadi S Rezaei S Hosseinifard H Dehnad A Shabaninejad H. The worldwide prevalence of anxiety in acquired immune deficiency syndrome patients: a systematic review and meta-analysis. Med J Islam Repub Iran. (2021) 35:101. 10.47176/mjiri.35.101 34956947 PMC8683796

[16] Lee KW Ang CS Lim SH Siau CS Ong LTD Ching SM. Prevalence of mental health conditions among people living with HIV during the COVID-19 pandemic: a rapid systematic review and meta-analysis. HIV Med. (2022) 23:990–1001. 10.1111/hiv.13299 35304829 PMC9111307

[17] Nouri E Moradi Y Moradi G. What is the global prevalence of depression among men who have sex with men? A systematic review and meta-analysis. Ann Gen Psychiatry. (2022) 21:38. 10.1186/s12991-022-00414-1 36096814 PMC9465955

[18] Zewudie BT Geze S Mesfin Y Argaw M Abebe H Mekonnen Z. A systematic review and meta-analysis on depression and associated factors among adult HIV/AIDS-positive patients attending ART clinics of Ethiopia: 2021. Depress Res Treat. (2021) 2021:8545934. 10.1155/2021/8545934 34721902 PMC8550854

[19] Smith A Breazeale S Goulet JL Vlahov D Justice AC Womack JA. A systematic review of risk factors for suicide among persons living with HIV (1996-2020). AIDS Behav. (2022) 26:2559–73. 10.1007/s10461-022-03591-y 35107660

[20] Du Zeying M Ashcroft T Kulkarni D Sawrikar V Jackson CA. Psychosocial interventions for depression delivered by non-mental health specialists to people living with HIV/AIDS in low- and middle-income countries: a systematic review. J Glob Health. (2022) 12:04049. 10.7189/jogh.12.04049 35976003 PMC9185189

[21] Yu Y Wang X Wu Y Weng W Zhang M Li J. The benefits of psychosocial interventions for mental health in men who have sex with men living with HIV: a systematic review and meta-analysis. BMC Psychiatry. (2022) 22:440. 10.1186/s12888-022-04072-1 35768860 PMC9241196

[22] Beard C Millner AJ Forgeard MJC Fried EI Hsu KJ Treadway MT. Network analysis of depression and anxiety symptom relationships in a psychiatric sample. Psychol Med. (2016) 46:3359–69. 10.1017/S0033291716002300 27623748 PMC5430082

[23] Borsboom D. Psychometric perspectives on diagnostic systems. J Clin Psychol. (2008) 64:1089–108. 10.1002/jclp.20503 18683856

[24] Borsboom D Cramer AOJ. Network analysis: an integrative approach to the structure of psychopathology. Annu Rev Clin Psychol. (2013) 9:91–121. 10.1146/annurev-clinpsy-050212-185608 23537483

[25] Choi KW Batchelder AW Ehlinger PP Safren SA O'Cleirigh C. Applying network analysis to psychological comorbidity and health behavior: Depression, PTSD, and sexual risk in sexual minority men with trauma histories. J Consult Clin Psychol. (2017) 85:1158–70. 10.1037/ccp0000241 29189032 PMC5724394

[26] Zhu Z Guo M Dong T Han S Hu Y Wu B. Assessing psychological symptom networks related to HIV-positive duration among people living with HIV: a network analysis. AIDS Care. (2022) 34:725–33. 10.1080/09540121.2021.1929815 34043459

[27] Wu Z Xu S Li J. The construction of mental health inventory for Chinese elderly. Chin J Clin Psychol. (2002) 10:1–3. 10.3969/j.issn.1005-3611.2002.01.001

[28] Yang Z. The construction and interpretation of multidimensional hierarchy of mental health. J Teacher Educ. (2022) 9:10–20. 10.13718/j.cnki.jsjy.2022.01.002

[29] Derogatis LR Melisaratos N. The brief symptom inventory: an introductory report. Psychol Med. (1983) 13:595–605. 10.1017/S0033291700048017 6622612

[30] Watson D Clark LA Tellegen A. Development and validation of brief measures of positive and negative affect: the PANAS scales. J Pers Soc Psychol. (1988) 54:1063–70. 10.1037/0022-3514.54.6.1063 3397865

[31] Lubeck DP Fries JF. Assessment of quality of life in early stage HIV-infected persons: data from the AIDS time-oriented health outcome study (ATHOS). Qual Life Res. (1997) 6:494–506. 10.1023/A:1018404014821 9330550

[32] Zhu Z. The Development of Integrated HIV/AIDS Symptom Management Model Under the HIV/AIDS Designated Hospital System in China, Shanghai: Fudan University (2018).

[33] Gajdoš P JeŽowicz T Uher V Dohnálek P. A parallel Fruchterman–Reingold algorithm optimized for fast visualization of large graphs and swarms of data. Swarm Evol Comp. (2016) 26:6. 10.1016/j.swevo.2015.07.006

[34] Zhu Z Xing W Hu Y Wu B So WKW. Paradigm shift: moving from symptom clusters to symptom networks. Asia Pac J Oncol Nurs. (2022) 9:5–6. 10.1016/j.apjon.2021.12.001 35528791 PMC9072174

[35] Parcesepe AM Filiatreau LM Ebasone PV Dzudie A Pence BW Wainberg M. Psychiatric comorbidity and psychosocial stressors among people initiating HIV care in Cameroon. PLoS One. (2022) 17:e0270042. 10.1371/journal.pone.0270042 35771857 PMC9246197

[36] Greene T Gelkopf M Fried EI Robinaugh DJ Lapid Pickman L. Dynamic network analysis of negative emotions and DSM-5 posttraumatic stress disorder symptom clusters during conflict. J Trauma Stress. (2020) 33:72–83. 10.1002/jts.22433 31433530

[37] Kaiser T Herzog P Voderholzer U Brakemeier E-L. Unraveling the comorbidity of depression and anxiety in a large inpatient sample: network analysis to examine bridge symptoms. Depress Anxiety. (2021) 38:307–17. 10.1002/da.23136 33465284

[38] Gebreyesus T Belay A Berhe G Haile G. Burden of fatigue among adults living with HIV/AIDS attending antiretroviral therapy in Ethiopia. BMC Infect Dis. (2020) 20:280. 10.1186/s12879-020-05008-4 32295546 PMC7161178

[39] Han S Hu Y Pei Y Zhu Z Qi X Wu B. Sleep satisfaction and cognitive complaints in Chinese middle-aged and older persons living with HIV: the mediating role of anxiety and fatigue. AIDS Care. (2021) 33:929–37. 10.1080/09540121.2020.1844861 33487030

[40] Hughes AM Campbell L Graham H Post F Chalder T. A biopsychosocial approach to hiv fatigue: a cross-sectional and prospective analysis to identify key modifiable factors. Behav Med. (2021) 47:205–13. 10.1080/08964289.2020.1712582 32078500

[41] Bedaso A Abraham Y Temesgen A Mekonnen N. Quality of sleep and associated factors among people living with HIV/AIDS attending ART clinic at Hawassa University comprehensive specialized Hospital, Hawassa, SNNPR, Ethiopia. PLoS One. (2020) 15:e0233849. 10.1371/journal.pone.0233849 32497153 PMC7272010

[42] Balthazar MS Webel A Gary F Burant CJ Totten VY Voss JG. Sleep and immune function among people living with human immunodeficiency virus (HIV). AIDS Care. (2021) 33:1196–200. 10.1080/09540121.2020.1770180 32482093 PMC7704700

[43] Solomon P O'Brien K Wilkins S Gervais N. Aging with HIV and disability: the role of uncertainty. AIDS Care. (2014) 26:240–5. 10.1080/09540121.2013.811209 23799874

[44] Furlotte C Schwartz K. Mental health experiences of older adults living with HIV: uncertainty, stigma, and approaches to resilience. Can J Aging. (2017) 36:125–40. 10.1017/S0714980817000022 28349859

[45] McElhiney MC Rabkin JG Daughters SB Timperlake EC Wainberg ML. Returning to work after fatigue treatment and counseling in HIV/AIDS. Work. (2019) 64:843–52. 10.3233/WOR-193046 31815724

[46] Lee KA Jong S Gay CL. Fatigue management for adults living with HIV: a randomized controlled pilot study. Res Nurs Health. (2020) 43:56–67. 10.1002/nur.21987 31612533

[47] Quigley A O'Brien K Parker R MacKay-Lyons M. Exercise and cognitive function in people living with HIV: a scoping review. Disabil Rehabil. (2019) 41:1384–95. 10.1080/09638288.2018.1432079 29376434

[48] Winston A Spudich S. Cognitive disorders in people living with HIV. Lancet HIV. (2020) 7:e504–13. 10.1016/S2352-3018(20)30107-7 32621876

[49] Alikhani M Ebrahimi A Farnia V Khazaie H Radmehr F Mohamadi E. Effects of treatment of sleep disorders on sleep, psychological and cognitive functioning and biomarkers in individuals with HIV/AIDS and under methadone maintenance therapy. J Psychiatr Res. (2020) 130:260–72. 10.1016/j.jpsychires.2020.07.043 32858346

[50] Zhu Z Hu Y Li H-W Bao M-J Zhang L Zha L-J. The implementation and evaluation of HIV symptom management guidelines: A preliminary study in China. Int J Nurs Sci. (2018) 5:315–21. 10.1016/j.ijnss.2018.08.005 31406842 PMC6626266

[51] Cioe PA Gordon REF Williams DM Kahler CW. The effect of increased physical activity on symptom burden in older persons living with HIV. AIDS Care. (2019) 31:1548–54. 10.1080/09540121.2019.1601675 30961364 PMC6764868

[52] Han S Pei Y Zhao R Hu Y Zhang L Qi X. Effects of a symptom management intervention based on group sessions combined with a mobile health application for persons living with HIV in China: a randomized controlled trial. Int J Nurs Sci. (2021) 8:370–9. 10.1016/j.ijnss.2021.07.002 34631986 PMC8488804

[53] Zhu Z Hu Y Xing W Guo M Zhao R Han S. Identifying symptom clusters among people living with HIV on antiretroviral therapy in China: a network analysis. J Pain Symptom Manage. (2019) 57:617–26. 10.1016/j.jpainsymman.2018.11.011 30465875

[54] Wenzel A. Basic strategies of cognitive behavioral therapy. Psychiatr Clin North Am. (2017) 40:597–609. 10.1016/j.psc.2017.07.001 29080588

[55] Moskowitz JT Addington EL Cheung EO. Positive psychology and health: well-being interventions in the context of illness. Gen Hosp Psychiatry. (2019) 61:136–8. 10.1016/j.genhosppsych.2019.11.001 31757566

[56] Dong N Chen WT Lu H Zhu Z Hu Y Bao M. Unmet needs of symptom management and associated factors among the HIV-positive population in Shanghai, China: a cross-sectional study. Appl Nurs Res. (2020) 54:151283. 10.1016/j.apnr.2020.151283 32425335 PMC7229465

[57] Sun W Lu H Bao M Zhang L. Experience of treatment naïve HIV patients receiving peer support: a qualitative study. Chinese J AIDS STD. (2020) 26:496–9. 10.13419/j.cnki.aids.2020.05.11

[58] Harris GE Larsen D. HIV peer counseling and the development of hope: perspectives from peer counselors and peer counseling recipients. AIDS Patient Care STDS. (2007) 21:843–60. 10.1089/apc.2006.0207 18240894

[59] Dutcher MV Phicil SN Goldenkranz SB Rajabiun S Franks J Loscher BS. “Positive Examples”: a bottom-up approach to identifying best practices in HIV care and treatment based on the experiences of peer educators. AIDS Patient Care STDS. (2011) 25:403–11. 10.1089/apc.2010.0388 21671756

[60] Øgård-Repål A Berg RC Skogen V Fossum M. Peer support in an outpatient clinic for people living with human immunodeficiency virus: a qualitative study of service users' experiences. BMC Health Serv Res. (2022) 22:549. 10.1186/s12913-022-07958-8 35468797 PMC9036816

[61] Webel AR. Testing a peer-based symptom management intervention for women living with HIV/AIDS. AIDS Care. (2010) 22:1029–40. 10.1080/09540120903214389 20146111 PMC3131156

